# Green Synthesis of pH-Responsive, Self-Assembled, Novel Polysaccharide Composite Hydrogel and Its Application in Selective Capture of Cationic/Anionic Dyes

**DOI:** 10.3389/fchem.2021.761682

**Published:** 2021-10-27

**Authors:** Nandita Srivastava, Anirban Roy Choudhury

**Affiliations:** ^1^ Academy of Scientific and Innovative Research (AcSIR), Ghaziabad, India; ^2^ Biochemical Engineering Research and Process Development Centre (BERPDC), Institute of Microbial Technology (IMTECH), Council of Scientific and Industrial Research (CSIR), Chandigarh, India

**Keywords:** polysaccharide hydrogel, stimuli-responsive, self-assembled, biodegradable, organic dye removal

## Abstract

Dyes are one of the most hazardous chemicals causing significant environmental pollution and affecting water quality. Majority of the existing methods for dye removal and degradation involve synthetic membranes and use of hazardous chemicals, further resulting in secondary pollution. The present study reports polysaccharide based novel composite hydrogel as biodegradable matrix for pH-responsive selective adsorption of cationic/anionic dyes. This membrane showed pH-responsive adsorption of methyl green (MG) and methyl orange (MO) with similar adsorption equilibrium, i.e., 315 and 276 mg g^−1^, respectively. Interestingly, selective adsorption at different pH has allowed separation of dye mixtures that holds incredible industrial importance for dyes recovery. The hydrogel matrix was able to completely separate MG, a model cationic dye at neutral pH from the dye mixture whereas, it was possible to remove 60% MO, a model anionic dye at acidic pH. Furthermore, comprehensive isothermal and kinetic studies of adsorption revealed that Freundlich isotherm describing the multilayer coverage and pseudo-second-order kinetics were followed. Thermodynamic studies indicated that the adsorption process was spontaneous and endothermic. In fact, the membrane was reusable for at least ten cycles and exhibited desorption efficiency of 80 and 60% for MO and MG, respectively, which may be further recycled to make the process environmentally sustainable. Overall, this study proposes an inexpensive, simple, biologically safe, and efficient adsorbent material for dye effluent treatment.

## Introduction

In recent years, waterbody safety issues have stimulated widespread apprehensions among the population (Rafatullah et al., 2010). Release of dye contaminated wastewater affects oxygenation of water bodies and harms aquatic life. This has become as cause of global environmental concerns and has drawn attention of researchers across the globe. Commercially available dyes are majorly used in textile, paper, tannery, plastics, and paints industries. However, regardless of their prodigious applications, organic dyes are non-biodegradable, allergic, carcinogenic, mutagenic, and toxic (Saghian et al., 2020). The complex aromatic groups present in these organic dyes are responsible for their low biodegradability which reduces the possibility of water recycling (Bazrafshan et al., 2014).

Dyes elimination through wastewater treatment is challenging and diverse treatment methods are employed for its removal. These approaches include biodegradation, membrane separation, chemical oxidation, photocatalysis, coagulation-flocculation, and adsorption (Li et al., 2017). Majority of these techniques are energy and cost-intensive. For example, chemical treatment of dyes would require high agitation and high temperature that may be further associated with sludge removal process (Arslan et al., 2016). On the other hand, physical methods like adsorption have been found to be simple, highly efficient, and economically feasible approach towards mitigating dye-containing effluents (Lei et al., 2013; Xiao et al., 2016). Numerous synthetic adsorbents based on activated carbon, zeolites, hydrogels, clay, etc., are known to remove toxic organic dyes from wastewater (Hu et al., 2018). However, these adsorbents have limitations in terms of resource recovery, purification, and reusability. Moreover, the chemical reagents used for cross-linking in synthetic hydrogels, such as N,N′-methylenebisacrylamide, and glutaraldehyde, are highly toxic and impose adverse health effects on living beings (Qi X. et al., 2018). Thus, their low or no biodegradability may cause secondary environmental pollution, one of the major concerns associated with the application of such adsorbents.

Natural polysaccharide-based materials are evolving as notable alternative adsorbents over synthetic ones for the removal of environmental effluents. Polysaccharide-based adsorbents are gaining prominence due to their biodegradable nature, non-toxic behavior, high binding affinity, easy and quick designing (El-Naggar et al., 2018). Polysaccharide-based hydrogels are three-dimensional polymeric networks that are suitable for water absorption in large quantities without undergoing dissolution. They are used to treat dye-polluted effluents due to their enhanced adsorption capacity, reusability, and smooth operation (Deng et al., 2013). Researchers have studied various adsorbents designed from natural polymers, for example, cellulose/chitosan hydrogel beads (Li et al., 2016), cellulose nanocrystals/alginate hydrogel beads (Mohammed et al., 2015), pullulan/polydopamine hydrogels (Su et al., 2020), etc. However, majority of these adsorbents are active only at a lower pH range, adsorbs single dye, and exhibit low dye retention capacities. In addition to the nature of the membrane, handling of dyes adsorbed on the membrane poses another problem. At present, normally the adsorbed dyes are degraded while removing them from wastewater or the adsorbent. The degradation of dyes may be carried out through photocatalytic degradation, use of nanoparticles, and other chemical methods and in all these cases, it have been reported to form by-products that cause additional toxicity and confines the reusability of dyes (Li et al., 2019). Therefore, all these issues gravely limit the applicability of existing adsorbents in dye containing wastewater treatment. The exploitation of highly efficient, biodegradable, and economical adsorbents with selective dye adsorption and desorption property may be a possible solution for treating wastewater with dye contaminants.

Stimuli-responsive hydrogels may be suitable alternative towards existing adsorbents and treatment methods. Stimuli can be provided by making alterations in the pH, ionic strength, and temperature of the hydrogel system. pH-responsive polysaccharide-based hydrogels have been studied extensively for their adsorption potential. Such hydrogels can react to changes in the ionic concentrations that can modulate their adsorption capacity. Furthermore, such charged polymers may interact electrostatically and form an interpenetrating self-assembled network. Electrostatic interaction can help to achieve dye adsorption and separation with an efficient single biodegradable membrane imperative for both dye reusability and recovery.

In the present work, we have prepared and investigated a novel, pH-responsive, self-assembled tri-composite hydrogel of chitosan, gellan, and κ-carrageenan (CH/GG/CR) for reusing and recovering toxic dyes from wastewater released by industries. The tri-composite hydrogel was formed without using any chemical cross-linkers. Further, the hydrogel swelling properties were assessed at different temperatures and pH to understand its adsorption capacity. Subsequently, the membrane was employed to selectively adsorb and desorb cationic and anionic dyes at different pH singly as well as in combination. This phenomenon has been demonstrated using methyl green (MG) and methyl orange (MO) as model dyes. Comprehensive adsorption kinetics, isotherms, and thermodynamic studies were performed to detect the time-dependent uptake and spontaneity of the adsorption reaction. The reusability of hydrogel was confirmed by studying the number of adsorption-desorption cycles. Overall, this study proposes an inexpensive, simple, biologically safe, and efficient adsorbent material for wastewater treatment and has made an effort towards zero dye effluent waste.

## Materials and Methods

### Materials

**FIGURE 1:**
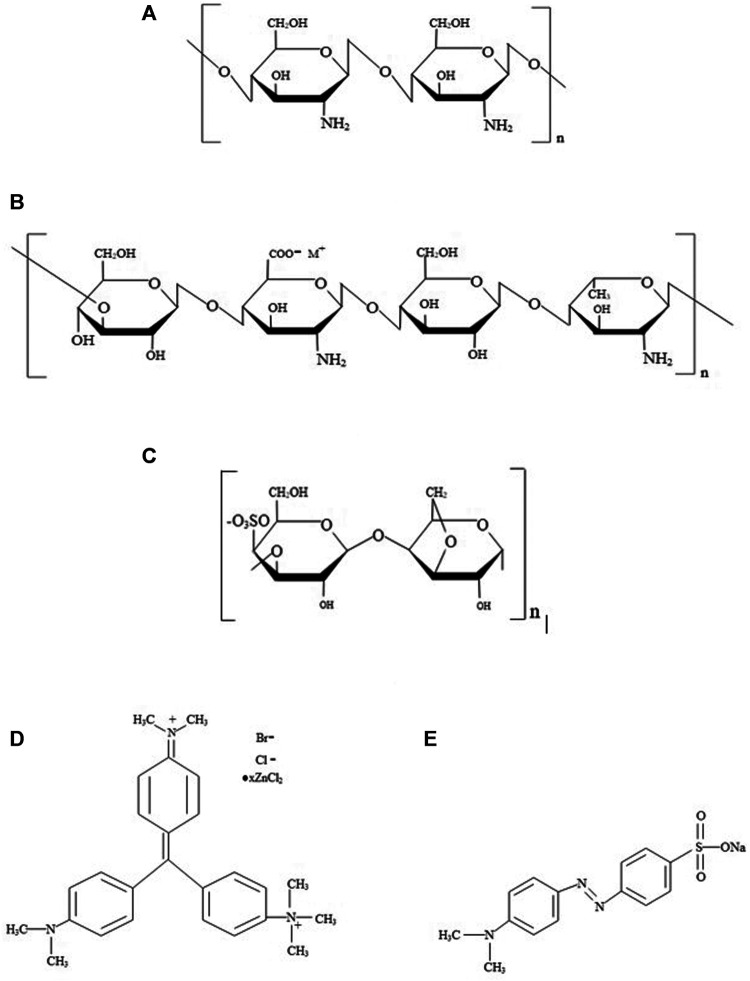
Structure of **(A)** Chitosan, **(B)** Gellan, **(C)** κ-Carrageenan, **(D)** Methyl Green and **(E)** Methyl Orange.

Commercially available chitosan having *M*_*w*_ = 3.8–20 kDa with the degree of deacetylation ≥75% (PCT0817, HiMedia, India), gellan gum of *M*_*w*_ = 500 kDa (Gelzan, G1910, Sigma-Aldrich, India), and κ-carrageenan having *M*_*w*_ = 788.7 Da (22048, Sigma-Aldrich, India) were utilized for hydrogel preparation. Potassium bromide (KBr) (Sigma-Aldrich, India) was used for the FT-IR analysis of hydrogel. Commercially available Methyl Green (MG) of *M*_*w*_ = 653.24 Da (HiMedia, India) and Methyl Orange (MO) of *M*_*w*_ = 327.34 Da (Merck, India) were used for dye adsorption and separation studies. The structure of polysaccharides and dyes are represented in Figure 1. Chitosan (CH) is a cationic linear polysaccharide composed of a linear chain constituting β(1→4)-d-glucosamine and N-acetyl-d-glucosamine units, derived from the partial deacetylation of chitin (de Oliveira et al., 2020). The gellan gum (GG) is an anionic polysaccharide composed of deacetylated α-l-rhamnose, β-d-glucose, and β-d-glucuronate units, respectively (Osmałek et al., 2014). Likewise, κ-carrageenan (CR) is an anionic sulfated polysaccharide containing alternating α (1–3)-d-galactose-4-sulfated and β (1–4)-3,6-anhydro-d-galactose units (Liu and Li, 2016). MG is a cationic triphenylmethane dye used as a colorant in the textile industries, and MO is an anionic dye that belongs to the azo group containing nitrogen (Alardhi et al., 2020).

### Preparation of Hydrogel

CH (1.0%, w/v) was prepared in 1.0% (v/v) acetic acid with continuous stirring at 600 rpm overnight at room temperature to obtain a pale yellow color viscous solution. GG and CR (1.5%, w/v) were prepared by dissolving weighed polysaccharides into preheated (90°C) deionized water while stirring until the homogenous viscous solution was obtained. To prepare a tri-composite hydrogel, aliquots of CH/GG/CR were mixed in ratio of 1:1:1 and allowed to form a three-dimensional polymeric network at room temperature. Similarly, bi-composites of these three polysaccharides were prepared by mixing them in equal proportions, i.e., CH/GG, CH/CR, and GG/CR, to compare its adsorption properties with the tri-composite CH/GG/CR hydrogel.

### Characterization of Tri-Composite Hydrogel

#### Fourier Transform Infrared (FT-IR) Spectroscopy

FT-IR was performed to understand the chemical linkages present in the composite hydrogels. FT-IR of sample was recorded by mixing lyophilized tri-composite hydrogel (1 mg) with KBr (99 mg) (Richa and Roy Choudhury, 2019a). The sample pellet was then subjected to FT-IR spectral analysis within the range of 4,000 to 400 cm^−1^ with a resolution of 1 cm^−1^. Additionally, infrared spectrum of individual polysaccharides was measured following the same procedure.

#### Dynamic Rheological Behavior

Rheological studies were conducted on a rheometer (MCR 102, Anton Paar, Austria) equipped with a Peltier plate system for accurate thermoregulation. The rheological tests were carried out using a parallel plate (PP) system (40 mm diameter) with a 1.0 mm set gap and temperature of 30°C (Richa and Roy Choudhury, 2019a). Flow curve was studied under the shear rate range of 0.001–1,000 s^−1^ to understand flow behavior of bi- and tri-composite hydrogel. An amplitude (γ) sweep was also performed to determine the linear viscoelastic region (LVR). The storage modulus (G′) and loss modulus (G″) were measured at an angular frequency (ω) ranging from 0.1 to 100 rad s^−1^ and strain amplitude of 0.1%.

#### Thermogravimetric Analysis

Thermogravimetric Analysis (TGA) was performed using the TGA/DSC I system (Mettler Toledo, Switzerland). Thermal stability of hydrogel was analyzed by exposing it to a temperature up to 650°C under the nitrogen atmosphere. The thermal stability was recorded as a weight loss percentage (Balasubramanian et al., 2018).

### Swelling Behavior of Tri-Composite Hydrogel

The swelling capacity of dehydrated tri-composite hydrogel was studied at different pH and temperatures through gravimetric analysis (Alonso et al., 2014). It was determined by immersing 400 mg dried hydrogel into 20 ml deionized water. Following incubation, excess water was wiped off from samples, and weight of swollen hydrogel was measured until equilibrium was reached. To determine the effect of temperature, swelling capacities at 30, 50, and 80°C were studied at pH 7. Similarly, swelling behavior of hydrogel was assessed in different pH solutions (2, 4, 7, 10, and 12) to study the effect of pH at room temperature. The pH of aqueous solution was adjusted using 1 M HCl and 1 M NaOH. All the experiments were performed in triplicates. The equilibrium swelling capacity percentage (SC%) of hydrogel was calculated using Eq. 1:

$$SC\%=\frac{W_{s}-W_{0}}{W_{0}}\times 100$$ (1)

where, *W*_*S*_ and *W*_*0*_ is the weight of the swollen and dry hydrogel, respectively.

### pH-Responsive Swelling of the Tri-Composite Hydrogel

Stimuli-responsive modulation of swelling capacity of the hydrogel was studied to understand its ability to respond towards pH alteration. For this purpose, pH of the aqueous medium was modulated by using dilute HCl solution (pH = 2) and phosphate-buffered solution (PBS) (pH = 7) (Wang et al., 2012). Initially, 400 mg dried hydrogel sample was swollen in a solution with pH 2 for 1 h and weight change was measured. Afterward, the gel was transferred to a solution with pH 7 for another 1 h and its weight was measured after drying off the excess water. This was repeated for three consecutive cycles by transferring hydrogel to alternate solutions every 1 h. The swelling capacity percentage was calculated according to Eq. 1.

### Adsorption Performance of Hydrogel

The dye adsorption potential of bi- and tri-composite hydrogel membranes were compared by immersing accurately weighed hydrogels in model ionic dyes, i.e., MG and MO solutions. For conducting batch isothermal studies, 20 mg dried hydrogel samples were immersed in 20 ml MG (pH∼7) and MO (pH∼2) solutions and kept at 150 rpm until adsorption equilibrium was reached. The initial dye concentrations varied from 100 to 400 mg L^−1^. Simultaneously, standard curve for dyes were plotted by measuring concentration of MG at 633 nm and MO at 465 nm through UV-Vis spectrophotometer (Hitachi U-2900) (Li et al., 2015; Murgai et al., 2020). The equilibrium quantity of dye adsorption was calculated as follows:

$$\;Qe=\frac{\;C_{0}-\;C_{e}}{m}\;\times \;V$$ (2)

where *Q*_*e*_ (mg g^−1^) is the capacity of dye adsorbed per gram hydrogel, *C*_*0*_ (mg L^−1^) and *C*_*e*_ (mg L^−1^) are the initial and equilibrium concentration of the dye, *V* (L) is the volume of the dye solution, and *m* (g) is the weight of dried hydrogel utilized in this study.

In order to perform adsorption kinetics experiments, a 20 mg dried CH/GG/CR hydrogel sample was added into 20 ml of MG (pH∼7) and MO (pH∼2) dye solution (100 mg L^−1^) at 30°C. After an interval of every 10 min, MG and MO concentrations of the solution were determined spectrophotometrically (Hu et al., 2018). Similarly, adsorption isotherm studies were conducted with different dye concentrations ranging from 100 to 400 mg L^−1^at 30°C. Additionally, the effect of temperature on dye adsorption was studied by performing experiments at three different temperatures, i.e., 30, 50, and 80°C (Richa and Roy Choudhury, 2019b).

### Selective Separation of Synthetic Dyes

It was important to understand the ability of CH/GG/CR hydrogel to separate the dyes from their mixtures for efficient recovery and zero dye release in the effluent. Firstly, mixture of dyes was prepared by adding 10 ml each of MG and MO having concentration 100 mg L^−1^ at pH 7 for each dye. Next, 20 mg of hydrogel sample was added into the dye mixture and agitated at 150 rpm for about 1 h at 30°C. The concentration of dye mixture was determined through a UV-Vis spectrophotometer (Agilent Technologies, Cary 60 UV-Vis) (Li et al., 2017). Next, the pH of solution was turned acidic (pH = 2), to check adsorption of remaining dye by submerging another hydrogel for additional 1 h at 30°C.

### Reusability of the Hydrogel

The hydrogel reusability was evaluated by performing adsorption-desorption cycles of organic dyes. For this, samples saturated with MG and MO were initially desorbed in deionized water by continuously stirring them at 150 rpm for 1 h (Hu et al., 2018). Subsequently, desorbed samples were exposed to individual dye solution (100 mg L^−1^). The concentrations of the MG and MO dye were measured as per the protocol mentioned in section “*Adsorption Performance of Hydrogel*”. Further, Eq. 3 was used to measure the dye removal percentage in each cycle (Meng et al., 2015).

$$Removal\;\%\;=\frac{\left(C_{0}-C_{t}\right)}{C_{0}}\times \;100$$ (3)

where *C*_*t*_ is dye concentration (mg L^−1^) at time *t*, respectively.

### Field Emission Scanning Electron Microscopy (FESEM) Analysis

The surface morphology of the tri-composite hydrogel was investigated through ultra-high-resolution field emission scanning electron microscopy (SU 8010 Series, Hitachi, Japan) at a voltage of 15 kV. For analysis, lyophilized hydrogel samples were fixed on conductive tape and coated with a gold-palladium layer (Hezaveh and Muhamad, 2013).

## Results and Discussion

### Preparation of Hydrogel

The tri-composite hydrogel was formed by self-assembly of chitosan, gellan, and κ-carrageenan via non-covalent electrostatic interactions among different functional groups present in those polysaccharides. This electrostatic interaction may be strengthened by Van der Waals forces and hydrogen bonding among the polymers. It may be commented that positively charged amino group (-NH_3_^+^) of CH interacts with the negatively charged sulfate (SO_4_^2−^) group of CR and carboxyl (-COO^−^) group of GG (Liang et al., 2018; de Oliveira et al., 2020), to form this self-assembled tri-composite hydrogel. Therefore, these interactions rendered use of chemical cross-linkers unnecessary for assembly of the tri-composite hydrogel. Previously, a report on chitosan/gellan gum composite hydrogel mentioned the establishment of polyelectrolyte complexes (PECs) due to oppositely charged groups in polysaccharides solution (de Oliveira et al., 2020). Such interactions improve the durability, mechanical strength, porosity, and structural homogeneity of hydrogel. In fact, this is the first report on a tri-composite hydrogel formed from these three natural polysaccharides.

### Characterization of Tri-Composite Hydrogel

#### Determination of Cross-Linking Among Hydrogel

**FIGURE 2:**
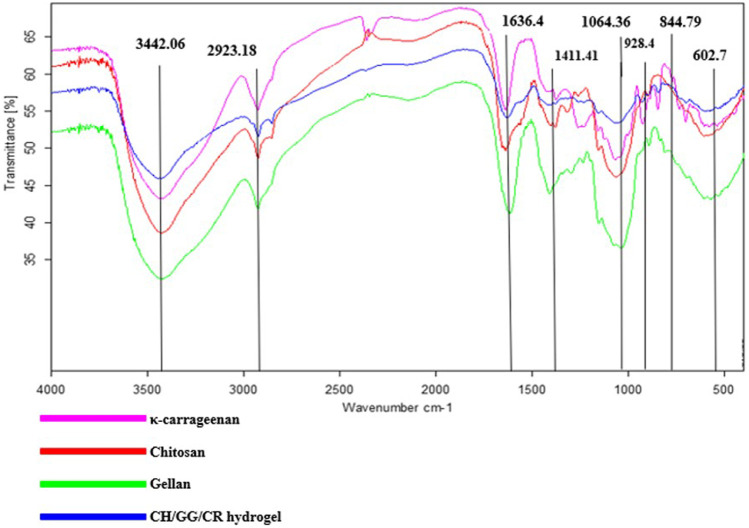
FT-IR spectra of CH/GG/CR tri-composite hydrogel.

FT-IR was performed to understand the chemical structure and interlinking among the polymer compounds (Figure 2). Infrared spectrum showed a characteristics band at 844.79 cm^−1^ due to the occurrence of d-galactose-4-sulphate being correlated to sulfate content of the CR, which provides it with a negative charge (Balasubramanian et al., 2018). Additionally, the peaks at 1,064.36 and 1,411.41 cm^−1^ were assigned to C-O linkages and symmetric COO^−^ stretching vibrations (Kudaibergenov et al., 2016; Tretinnikov et al., 2002). The presence of an infrared peak at 1,636.46 cm^−1^ corresponds to the glycosidic linkage in GG along with asymmetric COO^−^ stretching (Balasubramanian et al., 2018; Mohd Azam and Amin, 2017). The presence of carboxyl group imparts a negative charge to GG, which is imperative for electrostatic interactions. Also, a band in the region of 2,924.27 cm^−1^depicts C-H stretching (Patil and Netravali, 2019). Interestingly, a characteristic peak at around 3,442.06 cm^−1^ shows presence of O-H bonds (de Oliveira et al., 2020) and could be due to N-H stretching in CH, and this amino group is responsible for its positive charge (Khan et al., 2013). Furthermore, it was articulated from the adsorption spectra that CH/GG/CR tri-composite hydrogel unveiled slight shifts in peaks due to the hydrogel formation however still showed similarities with three individual polysaccharides. Hence, these observations confirmed successful crosslinking among constitutive polysaccharide network of tri-composite hydrogel. It is also noteworthy that after crosslinking, the native backbone structure of polysaccharide remained intact. However, there might be few free charged groups that did not participate in cross-linking and would further serve as active sites for dye adsorption.

#### Dynamic Rheological Behavior

**FIGURE 3:**
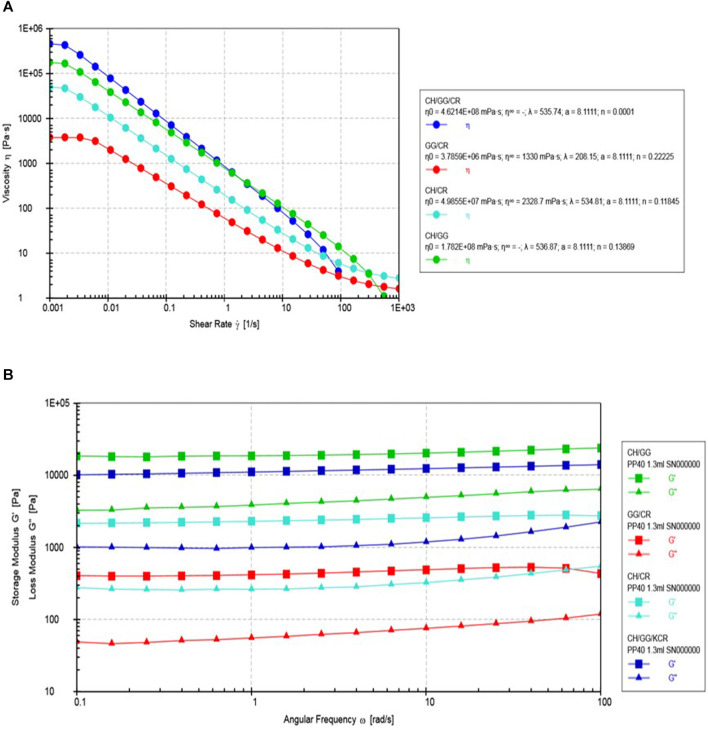
Rheological tests **(A)** viscosity (η) versus shear rate (γ), and **(B)** Storage modulus (G′) and loss modulus (G″) with angular frequency (ω).

Rheological characterization of hydrogel plays a significant role in gaining a deeper insight regarding the behavior of the material. The flow curve of the tri-composite hydrogel suggested that the synthesized gels were pseudoplastic in nature, shown in Figure 3A. The zero-shear rate viscosity (η_0_) of the hydrogels was found by fitting the Carreau-Yasuda model. The obtained values suggested that the tri-composite hydrogel had the highest η_0_ (4.62 × 10^8^ mPa s), followed by the bi-composite hydrogel composed of CH/GG. It must be noted that the bi-composite systems with chitosan as a component had higher viscosity. Therefore, it might be suggested that chitosan might play a crucial role in enhancing the viscosity of the hydrogel system. Earlier, Irimia et al. (2018) has mentioned the use of chitosan for enhancing viscosity of chitosan/PVA hydrogel (Irimia et al., 2018).

Moreover, it was observed that all the hydrogels exhibited similar linear viscoelastic region (LVR) with shear rates ranging from 0.08 to 0.1%. Using the obtained LVR, effect of frequency on storage modulus (G′) and loss modulus (G″) was studied by conducting a frequency sweep which is represented in Figure 3B. It is well acknowledged that G′ values reflect crosslinking density and elastic nature of the hydrogel system, whereas G″ represents its viscous nature (Qi X. et al., 2018). It was revealed from the frequency sweep that both G′ and G″ are independent of variation in angular frequency, which is a commonly observed trend in hydrogels. For instance, a previous report on xanthan/gellan/pullulan composite hydrogel also exhibited the same tendency (Kalia and Roy Choudhury, 2019). However, the G′ values were the highest for the tri-composite hydrogel and CH/GG bi-composite hydrogel. Much like the trend in the case of viscosity, G′ was higher for composites containing chitosan as a component. This can be attributed to the fact that hydrogels having higher viscosity generally have more intensive crosslinking amongst the components.

#### Determination of Thermal Stability

**FIGURE 4:**
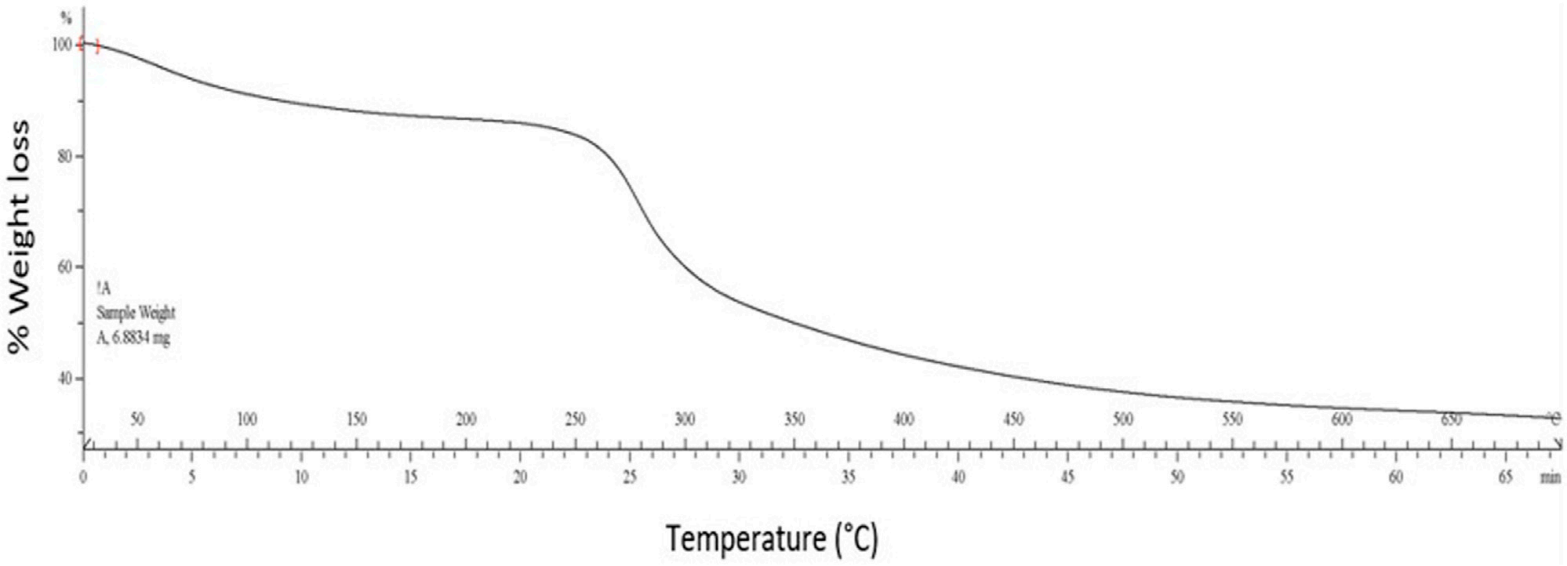
Thermogravimetric analysis of tricomposite hydrogel.

TGA is a thermal analysis method to detect the deviations in physical and chemical properties as a temperature rise function. The composite hydrogel’s TGA curve showed a decrease in weight in the three-step degradation pattern (Figure 4), as observed in most hydrogels. A marginal weight loss (∼8%) was initially observed from 40 to 110°C due to physically adsorbed water evaporation. Then, a nearly 50% decrease in weight was observed when the temperature was increased from 270 to 500°C, which may be associated with pyrolysis of polymers such as cleavage of bonds (Qi X. et al., 2018). Further, almost 20% loss in weight was detected above 500°C. Therefore, it is evident that the composite hydrogel remains relatively stable up to 270°C. It is essential to mention that the composite hydrogel resisted higher temperatures in contrast to the individual polysaccharides. Most of the previously reported composite hydrogels, such as cellulose/gelatin (Maity and Ray, 2017) and glucan/chitosan composite hydrogel (Jiang et al., 2019), followed a similar three-step degradation pattern.

### Swelling Behavior of Tri-Composite Hydrogel

#### Effect of Temperature

**FIGURE 5:**
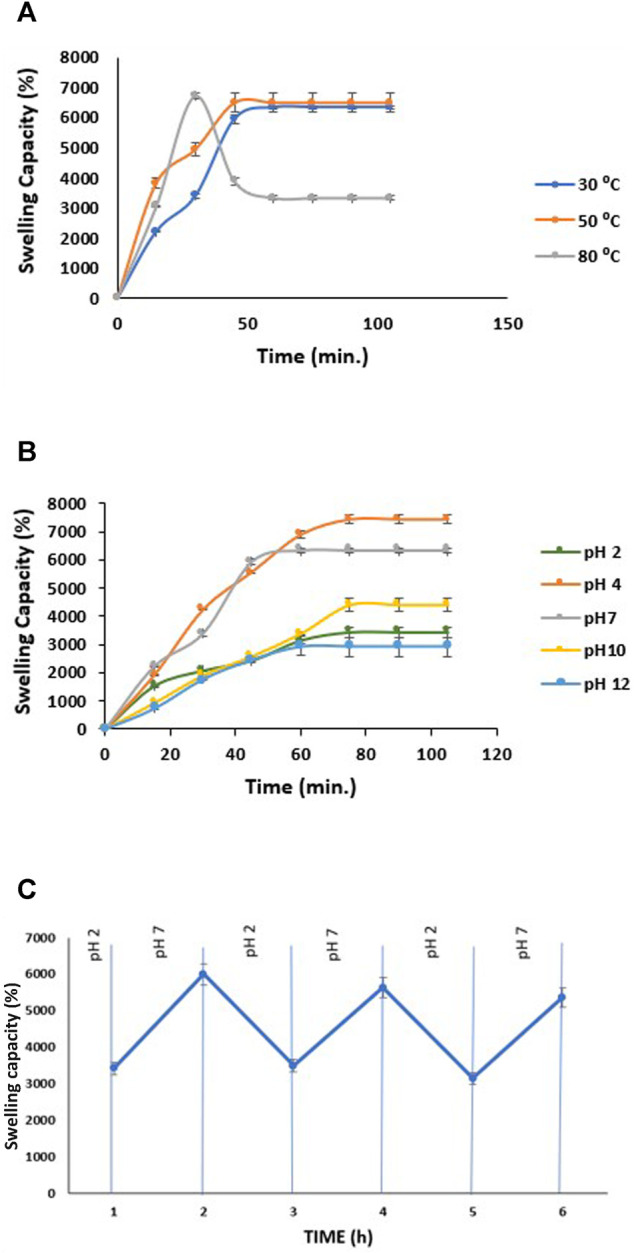
**(A)** Swelling capacity of CH/GG/CR hydrogel at a different temperature, **(B)** Swelling capacity of CH/GG/CR hydrogel at different pH, and **(C)** Pulsatile pH-dependent at 30°C with alternation of the swelling medium between HCl solution (pH = 2) and PBS (pH = 7); (*n* = 3, mean ± S.D.).

The swelling behavior of a material can be crucial in understanding its absorption capability. Here, we have studied the effect of temperature and pH on the adsorption capacity of tri-composite hydrogel, which will benefit adsorption and separation of dyes. The swelling kinetics of composite hydrogels was performed in water at three different temperatures, i.e., 30, 50, and 80°C (Figure 5A) until equilibrium was attained. The swelling capacity of tri-composite hydrogel increased gradually until 60 min at 30°C, after which it reached equilibrium at approximately 6,325%. Similarly, at 50°C, there was an initial rise for 45 min, afterward SC% became steady at 6,481%. A different trend of variation in swelling was observed at 80°C, where SC% increased to 6,713% within 30 min, after which values dropped to 3,332%, and the plateau region was attained further on. The crosslinking also opposes additional water movement by osmotic force due to an elastic retractive effect resulting in hydrogels to reach swelling equilibrium (Rizwan et al., 2017). Moreover, this equilibrium generates a balance amid the elastic retractive forces and the osmotic pressure of polymeric chains. The hydrogel displays a positive temperature response as it swelled faster with change in temperature from 30 to 80°C. The increase in temperature facilitates internal polymeric network expansion by enhancing chain mobility thereby, allowing high water uptake (Mah and Ghosh, 2013). However, at 80°C, swelling initially increased and gradually declined which may be due to disruption among strong polymer-polymer hydrogen bonding, causing the hydrogel to shrink and inhibit swelling.

#### Effect of pH

pH-sensitive swelling is accredited to charged hydrogels carrying different ionic groups. Various factors control the swelling capacity, such as ionic charge, degree of ionization, pK_a,_ or pK_b_ values of ionizable groups, hydrophilicity, and pH of the medium (Rizwan et al., 2017). Since the tri-composite hydrogel contains ionic polymers, they respond to low and high pH differently, resulting in different swelling capacities. Figure 5B shows that hydrogel has a higher swelling capacity at pH 4, followed by pH 7. In a highly acidic condition (pH ≤ 2), swelling capacity was low compared to pH 4 due to the alteration of carboxylic groups into the protonated acid form, enhancing the polymer hydrophobicity (Chang et al., 2011). As pH exceeds 4, ionization of some carboxylate groups and electrostatic repulsion among them resulted in an enhanced swelling capacity (Ju et al., 2001). Whereas, at high alkaline conditions (pH ≥ 10), counterions (Na^+^) may have a screen effect on charged groups, thus preventing electrostatic interaction (Llanes et al., 2020). This ultimately resulted in the lowest swelling ratio at pH 12.

### pH-Responsive Swelling of Tri-Composite Hydrogel

The reversibility in swelling capacity of hydrogel with alteration in pH of system was studied to understand stimuli-responsive behavior that can be further utilized for ionic dyes adsorption as different pH. In this regard, swelling capacity was found to be reversible between pH 2 and pH 7 (Figure 5C). At pH 7, hydrogel swells up to approximately 6,000%, whereas at pH 2 swelling capacity reduces to nearly 3,400% for three consecutive cycles. The internal osmotic pressure controls swelling of these hydrogels containing pH-responsive components that rises because of the ions and counterion movement. It is predicted that ion–counterion interactions balance the inner electrostatic repulsion among polymers (Khan et al., 2013). The porous structure of polymers forms capillary channels accountable for pH change response because of the natural diffusion of swelling medium into the polymeric matrix.

### Adsorption Performance of Hydrogel

**FIGURE 6:**
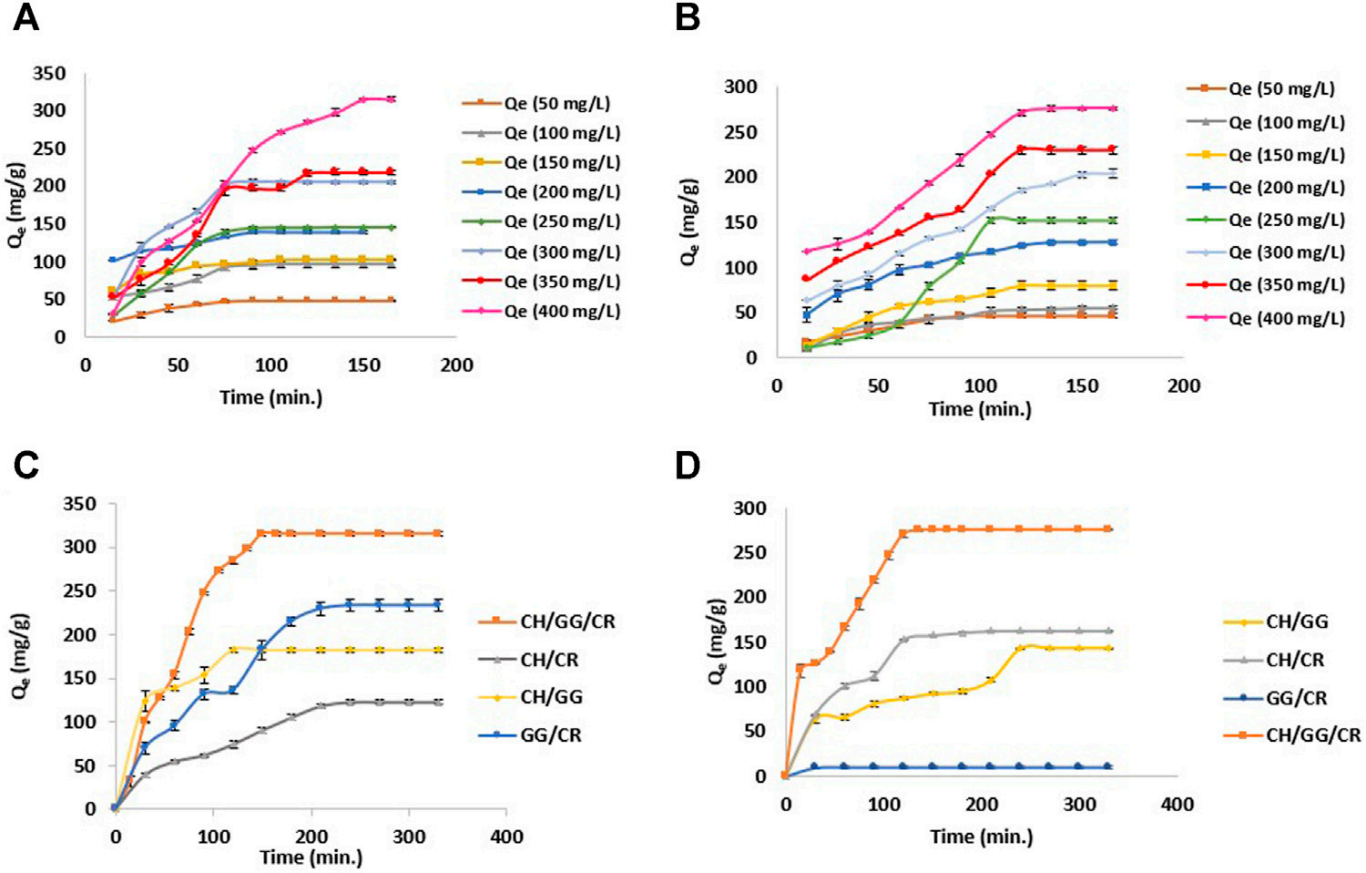
**(A)** Adsorption efficiency of CH/GG/CR hydrogel for MG, **(B)** Adsorption efficiency of CH/GG/CR hydrogel for MO, **(C)** Comparing adsorption efficiency of CH/GG/CR, CH/GG, CH/CR and GG/CR hydrogel for MG, and **(D)** Comparing adsorption efficiency of CH/GG/CR, CH/GG, CH/CR and GG/CR hydrogel for MO (*n* = 3, mean ± S.D.).

Primarily, the effect of dye concentration on adsorption of model ionic dyes MG and MO by CH/GG/CR hydrogel was observed at eight different concentrations ranging from 100 to 400 mg L^−1^ until equilibrium was reached. Initially, at 100 mg L^−1^, hydrogel exhibited similar adsorption capacity of 47.6 mg g^−1^ for MG and 45.02 mg g^−1^ for MO, respectively. However, it showed maximum adsorption capacity of 315 mg g^−1^ for cationic dye MG (pH 7) at 400 mg L^−1^ concentration in 150 min (Figure 6A). While, the hydrogel exhibited a maximum adsorption capacity of 276 mg g^−1^ for the anionic dye MO (pH 2) at 400 mg L^−1^ concentration in just 135 min (Figure 6B). This can be correlated with the swelling ability of hydrogel, which was low at pH 2 and high at pH 7 (Figure 5B). Later, the effect of time on adsorption of dyes on bi- and tri-composite hydrogels was compared. It was revealed from the furnished data that irrespective of dye, adsorption potential was highest in case of a tri-composite hydrogel. Among the synthesized bi-composite hydrogels, GG/CR exhibited higher adsorption for MG (230 mg g^−1^), CH/CR exhibited better adsorption for MO (157 mg g^−1^), while CH/GG showed average adsorption of both MG (183 mg g^−1^) and MO (143 mg g^−1^), respectively (Figures 6C,D). This can be attributed to a difference in charge of constitutive polysaccharides in the composite system. Since GG and CR are anionic polysaccharides, therefore they adsorb cationic dye MG in higher quantities. Similarly, composites containing CH, a positively charged biopolymer, exhibited higher adsorption potential for anionic dye, MO. Previously, clays (Satlaoui et al., 2019) and carbon nanotubes (Bahgat et al., 2012) have been utilized for MG dye adsorption with adsorption efficiencies of nearly 150 and 181.2 mg g^−1^, respectively. Recently, Wu et al. (2021) utilized halloysite nanotubes (HNTs) and chrysotile nanotubes (ChNTs) for MO dye adsorption with efficiency of 13.56 and 31.46 mg/g, respectively. The adsorption efficiency reported are lower as compared to our tri-composite hydrogel system. Since the adsorption capacity for both the dyes was highest in tri-composite hydrogel, it was used to conduct further studies.

### Adsorption Isotherm

The interactions among adsorbate molecules and an adsorbent surface can be explained through an adsorption isotherm. To examine the association between hydrogel and dye molecules for achieving equilibrium adsorption capacity, Freundlich and Langmuir isotherms were fitted to experimental adsorption data. The equations for both the isotherms are mentioned below:

$$Langmuir\;isotherm:\;\frac{C_{e}}{Q_{e}}=\frac{C_{e}}{Q_{m}}+\;\frac{1}{\;K_{L\;\;}Q_{m}}$$ (4)

$$Freundlich\;isotherm:\ln Q_{e}=\ln K_{F}\frac{1}{n_{F}}+\left(\ln C_{e}\right)$$ (5)

where *Q*_*e*_ (mg g^−1^) is concentration of dye adsorbed, *Q*_*m*_ (mg g^−1^) is monolayer coverage constant, *C*_*e*_ (mg L^−1^) is concentration of adsorbate, and *K*_*L*_ (L mg^−1^) is adsorption energy. *Q*_*m*_ and *K*_*L*_ are Langmuir constants, whereas *K*_*F*_ (mg g^−1^) and *n*_*F*_ (dimensionless) are Freundlich constant depicting the adsorption capacity and intensity. Langmuir isotherms elucidate the equal adsorption activation energy by each adsorbate molecule, which is being adsorbed onto the adsorbent’s surface. Thus, resulting in a monolayer coverage over specific sites in the adsorbent surface (Yang et al., 2016). Also, adsorption is considered irreversible since no migration of adsorbate occurs after adsorption. On the contrary, a Freundlich isotherm describes a heterogeneous adsorption system functional for the multilayer coverage and is a reversible process.

**FIGURE 7:**
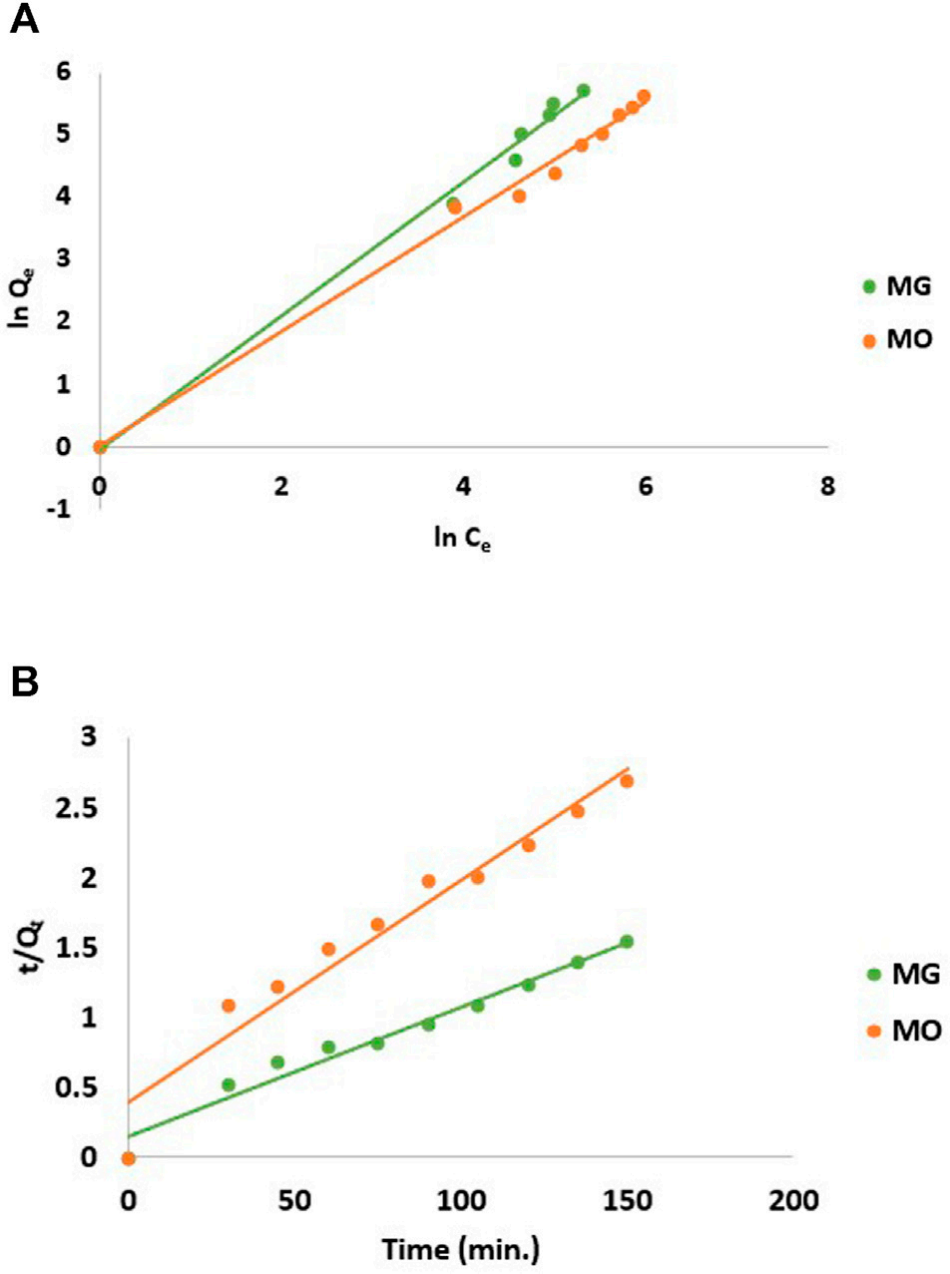
**(A)** Freundlich isotherm and **(B)** pseudo-second-order kinetics model for adsorption of dyes on CH/GG/CR hydrogel.

The linear regression was used to correlate experimental data with isothermic models to determine isotherm constants at 30°C (Table 1). The high correlation coefficient *R* ^2^ values for both the MG (*R* ^2^ = 0.99) and MO (*R* ^2^ = 0.99) dyes indicated that the Freundlich adsorption model shows a good fit with the data (Figure 7A). However, the Langmuir model poorly fits the adsorption progression with *R* ^2^ values of 0.88 and 0.41 for MG and MO, respectively (data not shown). Therefore, it was inferred that hydrogel surface sites had slightly variable adsorption energies for both the dyes and could adsorb dye molecules in a multilayer fashion. It is already known that process can be favorable with n_F_ > 1, linear with n_F_ = 1, and unfavorable with n_F_ < 1 (Kyzas et al., 2009). As the experimental data suggests that both the cationic and anionic dyes have n_F_ > 1. Thus, it can be concluded that the dye adsorption process here is favorable. Moreover, the adsorption space accommodates more than one layer of dye molecules, thereby allowing faster adsorption of dyes.

**TABLE 1:** Freundlich isotherm equilibrium constants for dye adsorption.

Dye	Q_e_ (mg g^−1^)	K_F_ [(mg g^−1^) (mg^−1^)^1/n^]	n_F_	*R* ^2^
MG	315.29	9.48	1.12	0.99
MO	276.05	1.004	1.08	0.99

### Adsorption Kinetics

Adsorption kinetic studies on dyes elucidate essential information on the evaluation of adsorption mechanisms. The kinetic data for MG and MO adsorption by hydrogel were fitted in accordance with the following equations:

$$\mathrm{pseudo-first-order:}\text{ln}\left(Q_{e}-Q_{t}\right)=\text{ ln}Q_{e}\;–\;k_{1}\text{t}$$ (6)

$$\mathrm{pseudo-second-order:}\;\frac{t}{Q_{t}}=\frac{1}{k_{2}Q_{e}^{2}}\;+\frac{t}{Qe}$$ (7)

Where *Q*_*t*_ (mg g^−1^) and *Q*_*e*_ (mg g^−1^) are adsorption capacity at a specified time “*t*” and equilibrium, respectively, and *k*_*1*_ (min^−1^) and *k*_*2*_ (g mg^−1^ min^−1^) are pseudo-first-order and pseudo-second-order adsorption rate constants (Junejo et al., 2019).

The determination coefficient (*R* ^2^) of pseudo-second-order model for MG and MO was 0.97 and 0.95, respectively. Therefore, dye adsorption onto hydrogel fits pseudo-second-order kinetic model represented in Figure 7B, which suggested removal from a solution is due to physicochemical interactions between the two phases. The values achieved for *k*_*2*_ and *Q*_*e*_ are reported in Table 2. Similarly, Qi X. et al. (2018) reported Salecan/poly (acrylamide-co-diallyldimethylammonium chloride) hydrogel for MO dye adsorption that followed pseudo-second-order model and best fitted with Freundlich isotherm (Qi X. et al., 2018).

**TABLE 2:** Pseudo-second-order kinetic and thermodynamics data for dye adsorption.

Dye	Kinetic parameters			Thermodynamic parameters					
	*R* ^2^	k_2_ (×10^−5^ g mg^−1^ min^−1^)	Q_e_ (mg g^−1^)	*R* ^2^	ΔH˚ (kJ mol^−1^)	ΔS˚ (kJ K^−1^ mol^−1^)	−ΔG˚ (kJ mol^−1^)		
							303K	313K	323K
MG	0.97	59.16	107.5	0.99	35.7	0.069	14.69	14.91	15.39
MO	0.95	81.8	61.3	0.91	11.32	0.012	15.04	12.5	12.73

### Adsorption Thermodynamics

The association between the process of adsorption and temperature is an imperative aspect to analyze as it helps interpret the thermodynamic factors of adsorption mechanism (Chen et al., 2012). The significant variations in Gibb’s free energy ΔG˚, enthalpy ΔH˚, and entropy ΔS˚ with temperature change was studied as per Eq. 8:

ΔG˚ = ΔH˚ − TΔS˚ (8)

However, it is already known that ΔG˚ = −RTlnK_c_, thus Eq. 9 can be revised as:

$$-RTlnK_{c}=\;\Delta H˚\;-\;T\Delta S˚$$ (9)

Where *T* is the temperature (*K*), *R* is universal gas constant (8.303 J mol^−1^ K), and *K*_*c*_ was analogous to Freundlich constant *K*_*F*_. The linear equation (Eq. 10) was utilized for plotting a graph between ln*K*_*c*_ and *1/T* to obtain values for thermodynamic parameters:

$$\text{ln }Kc=\;-\frac{\Delta H˚}{RT}+\;\frac{\Delta S˚}{R}$$ (10)

The slope and intercept of plot between ln *K*_*c*_ versus 1/T were considered for calculating values of thermodynamic parameters ΔH˚ and ΔS˚ (Kyzas et al., 2009). The values of ΔH˚ for hydrogels in case of MG (35.7 kJ mol^−1^) and MO (11.32 kJ mol^−1^) were positive (Table 2). This is suggestive of endothermic adsorption process. The negative values of ΔG˚ recommend spontaneity of process for dye molecules. ΔS˚ shows positive values for both MG (0.069 kJ K^−1^ mol^−1^) and MO (0.012 kJ K^−1^ mol^−1^) that correspond to amplified randomness at solid and liquid interface. Moreover, through the adsorption process, coordinated water molecules transferred by the dye molecules gain additional translational entropy, ensuing improved randomness in dye-hydrogel interactions (Unnithan and Anirudhan, 2001).

### Evaluation of Ionic Dyes Separation

**FIGURE 8:**
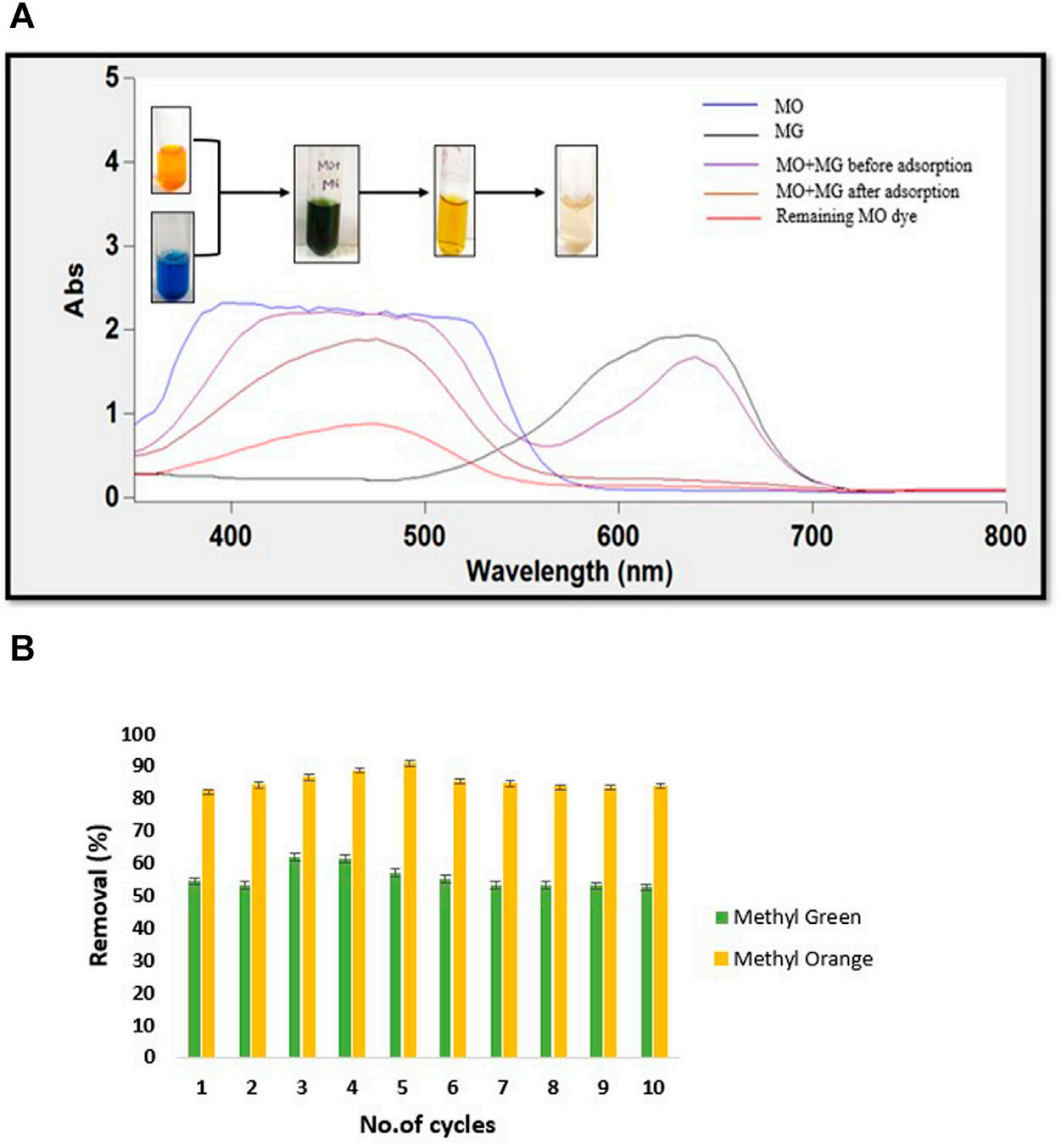
**(A)** UV-Vis spectra of before and after dye mixture separation by CH/GG/CR hydrogel and **(B)** Dye removal by hydrogel after cyclic adsorption-desorption (*n* = 3, mean ± S.D.).

Highly selective adsorbents could recover valuable chemicals and perform dye separation (Li et al., 2012). The individual capacity of hydrogel adsorption towards ionic dyes made it suitable for separation of dye mixture solutions. This was proven experimentally by preparing a mixture solution of MG and MO dye with a concentration of 100 mg L^−1^. The UV-vis spectra of the dye mixture showed two peaks at 630 and 460 nm, indicating presence of both the dyes in solution (Figure 8A). However, after the dye mixture (pH = 7) was passed through hydrogel membrane, only a single peak characteristic to MO was observed. The absorbance at the representative wavelength of MG was entirely negligible, suggesting its complete adsorption. It was also evident from the change in the mixture’s color from green to yellow after adsorption. Interestingly, the hydrogel could also remove nearly 60% of the remaining anionic dye after changing the pH of the mixture solution.

In fact, the digital images captured before and after dye mixture separation also conveyed the same information (Figure 8A). Earlier, Li et al. (2017) demonstrated the separation of only anionic dyes from a mixture of anionic and cationic dyes using PEE-Gel. However, to the best of our knowledge, there are no reports on using natural polysaccharide-based membranes to separate cationic and anionic dyes. Thus, it can be commented that novel CH/GG/CR hydrogel is first report on completely natural hydrogel to separate dyes selectively and minimize load of polluting dyes in final effluent.

### Reusability of the Hydrogel

The economic feasibility of a material can be determined by assessing recyclability of the employed adsorbent. For evaluating the reusability of synthesized hydrogel, saturated hydrogel was desorbed and again immersed in the aqueous dye solutions. It was detected that the hydrogel reserved its efficiency of dye removal for almost ten cycles during this entire procedure (Figure 8B). An initial rise followed by a minor decline in removal percent was observed, which may be due to strong binding strength of dye to hydrogel with each cycle. MO was removed in higher quantity (80% at pH 7) than MG (60% at pH 2). The hydrogel exhibited increased efficiency for retaining cationic dyes, and thus its removal was slightly difficult. Moreover, the well-cross-linked porous network structure of hydrogel was responsible for its incredible recyclability.

The adsorption capacity and reusability cycle of tri-composite hydrogel were compared with different polysaccharide-based adsorbents reported in the literature (Table 3). Upon comparing tri-composite hydrogel with previous reports, it was found that this hydrogel could overcome two main limitations of other adsorbents. Firstly, most adsorbents were synthesized using chemical cross-linkers (Qi C. et al., 2018; Pandey et al., 2020). Secondly, these composite hydrogels adsorbed either cationic or anionic dyes but could not adsorb and separate both the dyes simultaneously (Choudhury and Ray, 2018; Mohammed et al., 2015). Similar observations have been made in recent literature reports (Li et al., 2016; Qi X. et al., 2018), wherein cellulose/chitosan hydrogel beads and Salecan/poly (acrylamide-co-diallyldimethylammonium chloride) hydrogel were crosslinked through chemical reagents and adsorbed only anionic dyes. Likewise, cellulose nanocrystals/alginate hydrogel beads and Locust Bean Gum-cl-Poly(*N*,*N*-dimethylacrylamide) hydrogel were chemically crosslinked that adsorbed only cationic dyes, and it showed reusability for at least five cycles (Mohammed et al., 2015; Pandey et al., 2020). In contrast, the present study shows that hydrogel membrane was self-assembled, physically crosslinked, and adsorbed both cationic and anionic dyes at different pH levels. Moreover, the tri-composite hydrogel can be reused for at least ten cycles. Therefore, it may be economically and environmentally advantageous as compared to other adsorbents.

**TABLE 3:** Comparison of polysaccharide-based composite adsorbents for dye adsorption and reusability of synthetic dyes.

	Cross-linker	Dye adsorbed	Adsorption capacity (mg g^−1^)	Reusability cycles	References
Cellulose/Chitosan hydrogel bead	1-ethyl-3-methylimidazolium acetate ([Emim]Ac)	Congo red (anionic)	40	NA	Li et al. (2016)
Cellulose nanocrystals/alginate hydrogel beads	CaCl_2_	Methylene Blue (cationic)	256	5	Mohammed et al. (2015)
Graphene Oxide/Chitosan sponge	Self-assembled	Methylene Blue (cationic)	275	4	Qi et al. (2018a)
Salecan/poly (acrylamide-co-diallyldimethylammonium chloride) hydrogel	N,N′-methylenebisacrylamide	Methyl orange (anionic)	56.2	NA	Qi et al. (2018b)
Locust Bean Gum - cl -Poly(DMAAm) hydrogel	N,N′-methylenebisacrylamide	Brilliant green (cationic)	142	6	Pandey et al. (2020)
kappa-carrageenan (KC) and copolymers of poly (N-vinylpyrrolidone-co-acrylic acid) (CP)	Methylene bis-acrylamide (MBA)	Safranine T and Brilliant cresyl blue (cationic)	362.5 (Safranine T) and 398 (Brilliant cresyl blue)	5	Choudhury and Ray, (2018)
Pullulan/polydopamine hydrogels	Neopentyl glycol diglycidyl ether and Trimethylolpropane triglycidyl ether	Crystal violet and Methylene blue (cationic)	96 (Crystal violet) and 25.8 (Methylene blue)	NA	Su et al. (2020)
Chitosan/gellan/κ-Carrageenan hydrogel	Self-assembled	Methyl Green (cationic) and Methyl Orange (anionic)	315 (Methyl Green) and 276 (Methyl Orange)	10	Present study

### Analysis of Surface Morphology

**FIGURE 9:**
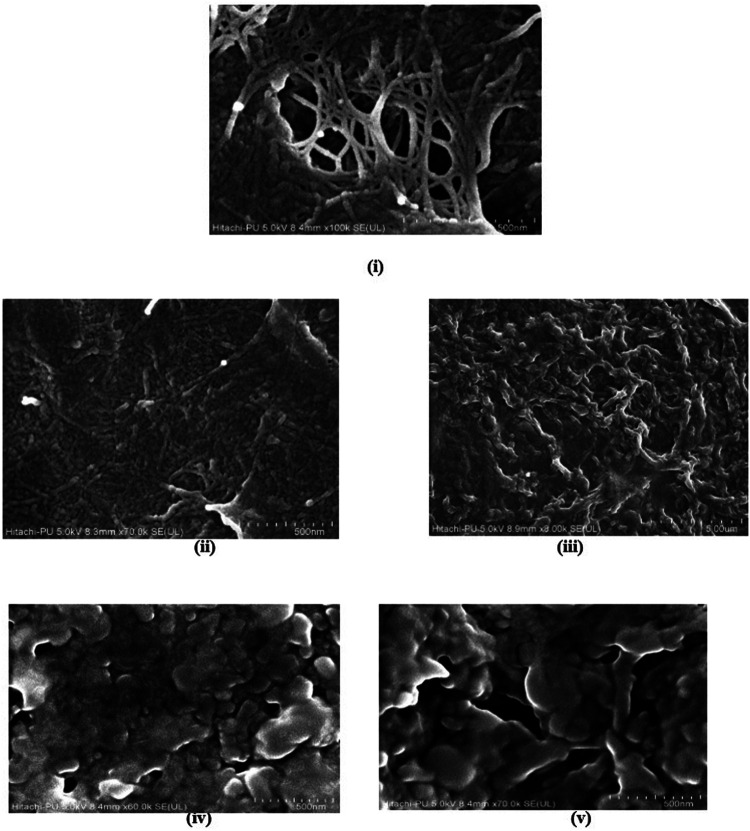
FE-SEM images of tri-composite hydrogel **(i)** before dye adsorption, **(ii)** after MG adsorption, **(iii)** after MO adsorption, **(iv)** after desorption of MG, and **(v)** after desorption of MO.

Understanding the surface morphology of hydrogel is essential for its utilization as an adsorbent. Therefore, FESEM analysis was performed to visualize the detailed hydrogel structure (Figure 9). The micrograph of sample demonstrated that tri-composite hydrogels have a rough and corrugated surface. The mesh-like internal structure, called microchannels signifies an interconnected porous surface that would ultimately assist in adsorption and transport of water and dyes. These microchannels are formed as a result of crosslinking among the polymers (Bashir et al., 2018). An earlier report on FESEM images of karaya gum-g- poly (acrylic acid) hydrogels also showed the porous surface that facilitates water and drug exchange (Bashir et al., 2018). A similar electron micrograph was reported for a super-adsorbent nanocomposite hydrogel that revealed a loose and porous surface for the transportation of water and dyes (Hu et al., 2018). Additionally, the surface morphology of hydrogel after dye adsorption and desorption for both MG and MO are represented in Figure 9, which suggested that after adsorption of dyes, the hydrogel surface becomes non-porous as pores remain clogged with dyes. Interestingly, after desorption, there was a significant difference in the surface morphology of hydrogel adsorbing MO and MG. In the case of MO, hydrogel shows a more porous surface than MG, which may be attributed to the difference in removal percent as mentioned above.

### Mechanism of Dye Adsorption and Desorption

**FIGURE 10:**
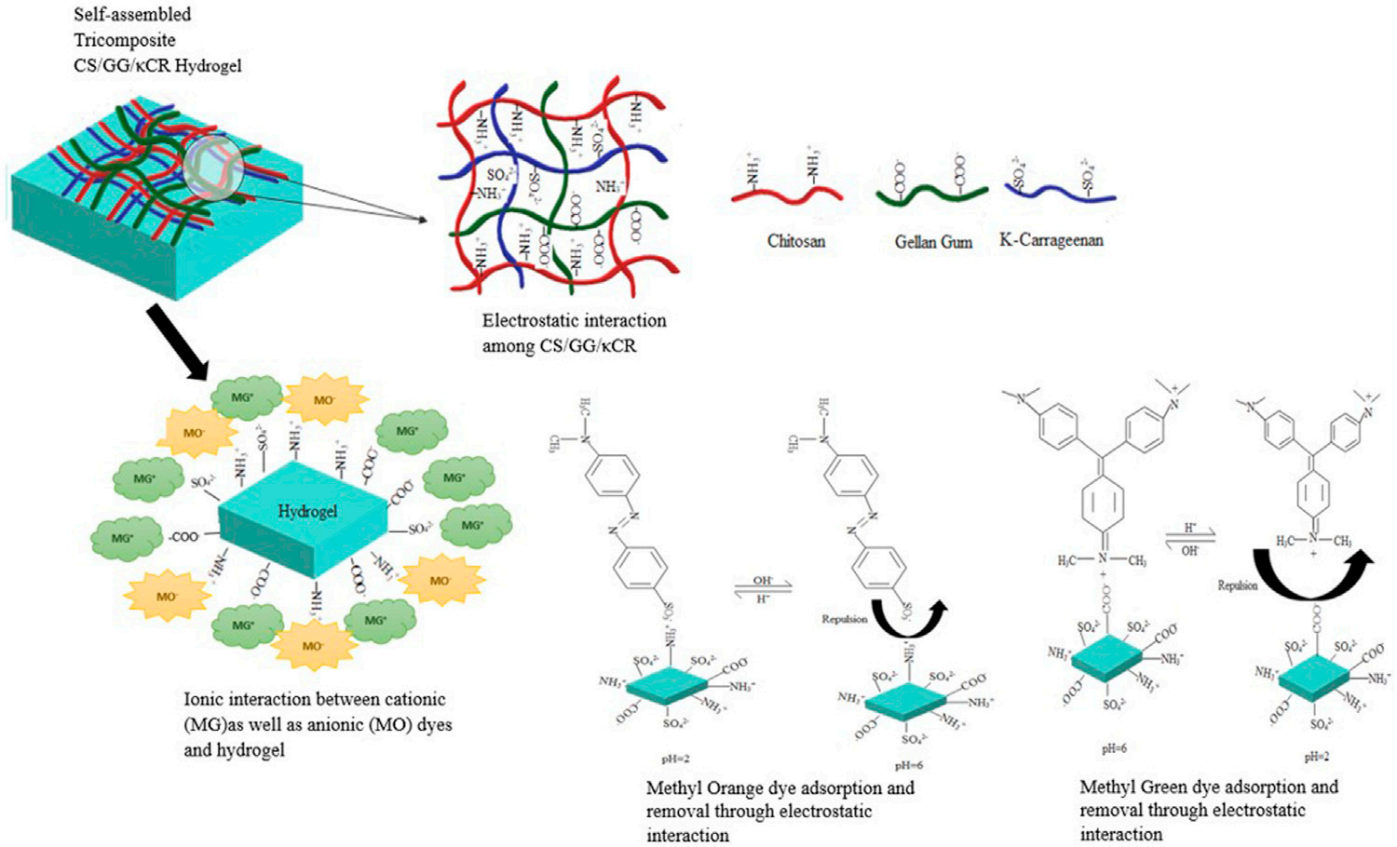
Schematic illustration of self-assembled tri-composite hydrogel along with the proposed mechanism for dye adsorption and desorption.

The proposed mechanistic scheme of dye adsorption has been shown in Figure 10. The advantage of using this polyelectrolyte hydrogel is that it allows adsorption of both dyes at different pH levels. The pH-responsive hydrogel generally contains both acidic and basic groups in polymeric matrix. It can be proposed that in acidic solutions, the surface charge on adsorbent is positive. Therefore, it is advantageous for adsorption of anionic dye due to enhanced electrostatic interactions. This interaction occurs between positively charged protonated amino group (NH_3_^+^ in chitosan) on hydrogel surface and negatively charged sulphate group (SO_3_^−^) on anionic (MO) dye. At high pH, surface of hydrogel containing negatively charged deprotonated groups (COO^−^ in gellan gum and SO_4_^2-^ in κ-carrageenan) interacts with two positive charges present on cationic (MG) dye (Figure 10) (Li et al., 2017). Simultaneously, few secondary interactions among the hydrogel and dyes (Van der Waals forces, hydrogen bonds, and porous networks) allowed dye molecules to be captured successfully through the adsorption mechanism (Ilgin et al., 2020). The possible reason for this high adsorption capacity of hydrogel for cationic dye is the presence of two anionic polysaccharides in polymeric network, whereas adsorption capacity for anionic dye was low due to the presence of only a single cationic polysaccharide in hydrogel matrix.

## Conclusion

The present work reports a novel facile method for green synthesis of tri-composite hydrogel to remove toxic organic dyes from industrial effluents. The hydrogel was self-assembled and formed without any chemical cross-linkers. Polyelectrolyte nature of the hydrogel allowed adsorption of cationic (MG = 315 mg g^−1^) and anionic (MO = 276 mg g^−1^) dyes at different pH. The dye adsorption process followed pseudo-second-order kinetics and best fitted with Freundlich isotherm model. Moreover, separation efficiency of hydrogel for cationic dye was almost 100% at pH 7. In contrast, it adsorbed more than 60% of the anionic dye from dye mixture on changing pH to acidic level (pH 2). It has reusability for at least ten cycles, thereby making it cost-effective. To date, this is the first report of self-assembled polysaccharide-based pH-responsive tri-composite hydrogel for selective adsorption of both ionic dyes using a single-membrane system, making an effort towards zero dye waste. This research work reports a durable, biodegradable, and highly efficient adsorbent material that can be effectively used for wastewater treatment.

## Data Availability

The original contributions presented in the study are included in the article/Supplementary Material, further inquiries can be directed to the corresponding author.
